# Clinical Outcomes of Surgical Treatment for Pyogenic Spondylitis According to Surgical Approach

**DOI:** 10.3390/jcm15103843

**Published:** 2026-05-16

**Authors:** Sung-Woo Choi, Chi Young An, Seung Lim Baek, Min Jung Baek, Jae Chul Lee, Hae-Dong Jang, Byung-Joon Shin

**Affiliations:** 1 Department of Orthopaedic Surgery, Soonchunhyang University Hospital, Seoul 04401, Republic of Korea; 132523@schmc.ac.kr (C.Y.A.); 135735@schmc.ac.kr (S.L.B.); jlee@schmc.ac.kr (J.C.L.); khaki00@schmc.ac.kr (H.-D.J.); schsbj@gmail.com (B.-J.S.); 2 Department of Obstetrics and Gynecology, CHA University College of Medicine, Seoul 06135, Republic of Korea; goodgood75@naver.com

**Keywords:** pyogenic spondylitis, anterior with or without posterior approach, posterior only approach, radiologic outcome

## Abstract

**Background/Objectives:** The access route to the surgical treatment of pyogenic spondylitis is a critical issue. However, no published clear guidelines are available about the access route for the surgical treatment of pyogenic spondylitis. The objective of this study was to compare clinical outcomes according to surgical treatment approach (anterior approach with or without posterior approach versus posterior only approach) of pyogenic spondylitis. **Methods:** This study included patients who underwent surgical treatment for pyogenic spondylitis. These patients were divided into two groups according to surgical treatment approach: anterior approach with/without posterior approach (anterior group) and posterior-only approach (posterior group). Their baseline data, treatment failure, radiologic outcome, and ambulatory ability were compared. **Results:** Demographic data and clinical characteristics were similar (*p* > 0.05) between the two groups. Baseline radiologic characteristics of infection, including infected spinal level, number of infected segments, presence of epidural abscess, and vertebral body collapse, were also comparable between the two groups. The number of unplanned surgeries was less in the anterior group than in the posterior group (*p* < 0.05). Approach-related complications tended to occur more often in the anterior group, although the difference was not statistically significant (*p* = 0.067). There was no significant difference in radiologic outcome or final ambulation ability after surgery between the two groups (*p* > 0.05). **Conclusions:** Anterior approach with or without posterior approach offers fewer treatment failure rates, particularly regarding revision surgeries, than a posterior-only approach. Thus, an anterior approach with or without a posterior approach should be used when planning surgical treatment for pyogenic spondylitis.

## 1. Introduction

Spondylodiscitis, also referred to as vertebral osteomyelitis, is a rare but potentially life-threatening infection involving the vertebral bodies and intervertebral disc, accounting for approximately 2–7% of all osteomyelitis cases [1]. Owing to its close anatomical relationship with the spinal cord and nerve roots, the disease frequently presents with neurological manifestations, including back pain, motor weakness, and sensory deficits in the lower extremities [2]. If left untreated, the infection may progress to vertebral body destruction with kyphotic deformity, spinal instability, and neural compression. Severe neurological deficits, including paralysis, may occur due to direct compression by collapsed bony structures or adjacent abscess formation. These complications are associated with prolonged hospitalization, increased rates of revision surgery, and higher mortality [3].

Conservative treatment with immobilization and systemic administration of antibiotics is initially recommended for patients [4]. Surgery may be considered if the patient suffers from intolerable severe pain, neurological deficits, and damage to spinal stability [5]. Surgical treatment is absolutely indicated in patients with spinal cord or cauda equina compression with progressive neurological deficits [6]. Emergency surgical decompression should be considered for such patients. Relative indications for surgery include failed conservative treatment, progressive spinal deformity with biomechanical instability, or selected cases requiring surgical debridement and tissue diagnosis [7].

Several strategies have been described for the surgical management of spinal infections, including an anterior approach and posterior approach. Traditionally, thorough and radical debridement of all infected or necrotic tissues is mandatory. An anterior approach provides direct access to this infected focus, enabling more thorough debridement of the disc space, endplates, and surrounding necrotic tissue, which is inherently difficult to achieve through a posterior-only corridor. It is convenient for debriding infection and reconstructing stability [6]. However, some patients with cardiovascular and respiratory diseases might experience more complications when an anterior approach is used [5]. In such cases, a posterior-only approach is convenient for drainage of abscess and instrumentation of posterior implants. Recently, various authors have obtained promising results with posterior instrumentation and fusion or even minimal invasive instrumentation [8].

Although the surgical approach of pyogenic spondylitis is a critical issue, currently no published clear guidelines are available about the access route in surgical treatment for pyogenic spondylitis. Thus, the objective of this study was to investigate clinical outcomes according to the surgical approach (anterior approach with or without posterior approach versus posterior only approach) of pyogenic spondylitis.

## 2. Materials and Methods

This retrospective cohort study was approved by our Institutional Review Board (IRB 2023-09-011). An institution-based electronic registry database for discharge diagnosis was searched to retrieve all cases of ‘infectious spondylitis’ and ‘vertebral osteomyelitis’ from January 2010 to December 2021 to identify those with pyogenic spondylitis who underwent surgical treatment in Soonchunhyang University Seoul Hospital. Each case was manually reviewed. In principle, spinal infections were treated in multidisciplinary ways. Infectious medicine, orthopedic surgery, neurosurgery, and radiology specialists discussed and decided the treatment policy. After surgeries, decision-making on antibiotic administration, type of antibiotic, duration of use, and failure of treatment were judged by the multidisciplinary team. We analyzed baseline data such as age, sex, smoking, body mass index (BMI), comorbidity, and ASA (Table 1).

### 2.1. Patient Selection

**Figure 1:**
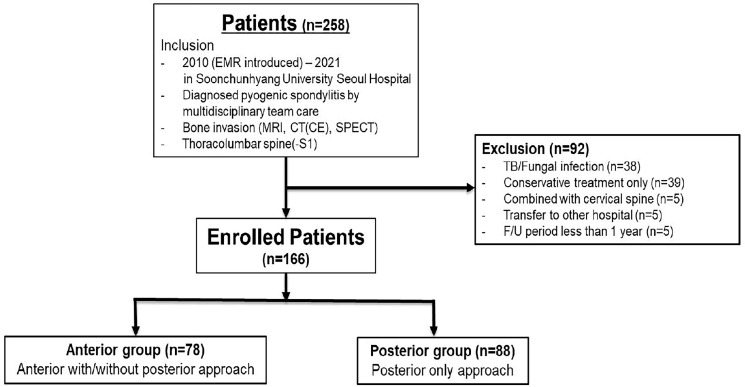
Flow chart showing the selection of study subjects with inclusion and exclusion criteria. EMR = Electronic medical record. MRI = Magnetic resonance imaging. CT (CE) = Computed tomography (Contrast enhanced). SPECT = Single Photon Emission Computed Tomography. TB = Tuberculosis. F/U = Follow-up.

(1) Patients diagnosed between 2010, when the electronic medical record system was introduced, and 2021; (2) patients who were cared for by a multidisciplinary team; (3) patients whose MRI, CT(CE), SPECT, or other radiologic study confirmed pyogenic spondylodiscitis with combined disc and vertebral body involvement; (4) infection involving the thoracolumbar spine (T1–S1). Exclusion criteria were: (1) those who had tuberculous or fungal infection, (2) patients who did not receive surgery (conservative treatment only), (3) patients whose infection involved the cervical spine, (4) patients who were transferred to other hospitals, or (5) patients whose follow-up period was less than one year. Included patients were classified into two groups according to treatment approach for pyogenic spondylitis: anterior approach with or without posterior approach (anterior group) and posterior only approach (posterior group) (Figure 1).

### 2.2. Definition

Primary infection was defined as pyogenic spondylitis occurring without prior spinal surgery. Secondary infection was defined as infection developing after previous spinal surgery [9]. Secondary infections were further classified according to whether metallic instrumentation had been used during the prior procedure. Stage of surgery was analyzed only for patients with an anterior approach along with a posterior approach. One-stage surgery was performed on the same day, while two-stage surgery with an interval between anterior surgery and posterior surgery. Baseline severity of infection was additionally evaluated using radiologic parameters. Infected spinal level was determined according to the main pathological focus of infection. In patients with multilevel involvement, the level corresponding to the dominant infected lesion was used for classification (e.g., extensive involvement from T10 to S1 categorized as lumbar spine if the main lesion was located in the lumbar region). The number of infected segments was defined as the total number of involved spinal levels identified on MRI or contrast-enhanced CT. Epidural abscess was recorded as present or absent based on MRI findings. The degree of anterior vertebral body collapse was evaluated on sagittal MRI or CT images. The anterior height of the infected vertebral body was compared with the average anterior height of the adjacent intact vertebrae above and below the involved level. Vertebral body height loss was categorized as <50% or ≥50%. Causative microorganisms were identified using tissue or blood culture. When we could gain access to the wound or treat it with surgery, we performed cultures for samples obtained from surgical sites. If culture results were different, tissue culture results were preferred. Hospital days after surgery refers to the period from the day of surgery for pyogenic spondylitis treatment to discharge. Total hospital days refers to the period from the day of admission to discharge. Unplanned surgery was defined as any reoperation performed after the index surgery for pyogenic spondylitis due to persistent infection (e.g., residual pus or sustained elevation of inflammatory markers) or mechanical complications such as implant loosening [10]. Radiologic outcome was determined by postoperative one-year X-ray. Lumbar instability was defined as (1) more than 8 mm of translation or more than 10° of angulation of motion (L1/2-L4/5) or (2) more than 4 mm of translation or 20° of angulation of motion (L5/S1) compared to adjacent motion segment instability in flexion-extension view [11]. The sagittal vertical axis was measured as the horizontal distance between a plumb line drawn from the center of the C7 and a line drawn from the center of C7 to the posterior superior corner of S1 [12]. Lumbar lordosis was the angle between the upper line drawn at the superior endplate of L1 and the lower line at the superior endplate of the sacrum [13]. An abnormal finding for lumbar lordosis was defined as above 80 degrees or below 20 degrees. Ambulatory ability was investigated by telephone to patients or family and assessed according to Koval’s classification (grade I: independent community ambulator; grade II: community ambulator with a cane; grade III: community ambulator with walker/crutches; grade IV: independent household ambulator, grade V: household ambulator with a cane; grade VI: household ambulator with walker/crutches; grade VII: nonfunctional ambulator) [14].

### 2.3. Surgical Technique

The standard anterior surgical management for infectious spondylitis involves thorough debridement of infected and necrotic tissues, anterior radical debridement, and interbody fusion via a lateral retroperitoneal approach, with or without posterior instrumentation. The patient is placed in the lateral decubitus position, and the retroperitoneal space is accessed through sequential dissection of the abdominal wall muscles. Following mobilization of the psoas muscle, the infected intervertebral disc and granulation tissue were completely removed. To restore anterior column stability, structural bone grafting using autologous and/or allogenic materials was performed. Subsequently, patients were repositioned to the prone position for posterior stabilization using bilateral pedicle screws and rods. Decompressive laminectomy was selectively performed during the posterior procedure only in patients presenting with neurological deficits secondary to epidural compression.

In posterior-only surgical management of spinal infections, laminotomy or laminectomy is performed to effectively remove purulent material and infected granulation tissue. This decompressive procedure alleviates neural compression caused by epidural abscess formation or inflammatory tissue, thereby promoting neurological recovery and facilitating infection control.

### 2.4. Statistical Analysis

Statistical analyses were performed using REX statistical software (version 3.7.1.0; RexSoft Inc., Seoul, Republic of Korea). Continuous variables were expressed as mean ± standard deviation and compared between groups using the independent *t*-test. Categorical variables were presented as frequencies and percentages and compared using the chi-square test or Fisher’s exact test, as appropriate.

To evaluate factors associated with clinical outcomes, generalized linear regression was used to estimate odds ratios (ORs) with 95% confidence intervals (CIs). A two-sided *p* value < 0.05 was considered statistically significant.

## 3. Results

A total of 258 patients met our inclusion criteria. Of these, those with tuberculous spondylitis (38 cases), patients managed with conservative treatment alone (39 cases), patients with cervical spine involvement (5 cases), those transferred to other hospitals (5 cases), and those with a follow-up period of less than one year (5 cases) were excluded. Ultimately, 166 patients were included in this study.

There were 97 (58.4%) women and 69 (41.6%) men, and the mean age was 67.0 years (range, 21–91 years). Of the 166 patients, 78 were operated on via an anterior approach with or without a posterior approach, and 88 underwent a posterior-only approach.

Demographic characteristics were not significantly different between the two groups. Age was 66.9 ± 12.2 years in the anterior group and 67.2 ± 12.2 years in the posterior group. In the anterior group, 61.5% were male and 38.5% were female, whereas in the posterior group, 55.7% were male and 44.3% were female. There were no statistically significant differences in smoking status, body mass index (BMI), or comorbidities, including hypertension, diabetes mellitus, liver cirrhosis, hemodialysis, and malignancy. The American Society of Anesthesiologists (ASA) physical status classification was also similar between the two groups (Table 1).

There was no significant difference in infection type (primary or secondary) either. In the anterior group, one-stage operation had 26 (36.6%) cases and two-stage surgery had 45 (64.0%) cases (Table 2). There was no significant difference in the level of infection in the spine between the two groups (*p* = 0.9534). Most cases involved the lumbar spine (L2–L5) in both groups (65.38% in the anterior group and 63.64% in the posterior group). There was also no significant difference in the number of infected segments between the groups (*p* = 0.5448). In both groups, single-segment involvement was the most common (66.7% in the anterior group and 72.7% in the posterior group), followed by two-segment involvement (20.5% in the anterior group and 19.3% in the posterior group). Involvement of three or more segments was less frequent in both groups. There was also no significant difference in the presence of epidural abscess between the two groups (*p* = 0.3085). Epidural abscess was identified in 39 patients (50.0%) in the anterior group and 52 patients (59.1%) in the posterior group. Similarly, the degree of anterior vertebral body collapse did not differ significantly between the two groups (*p* = 0.89). Collapse ≥ 50% was observed in 10 patients (12.8%) in the anterior group and 13 patients (14.8%) in the posterior group (Table 3). There were many kinds of microbes detected by blood or tissue culture. No growth was the most common result (38.46% in the anterior group and 43.18% in the posterior group). There was no statistically significant difference in the species of cultured microbes between the two groups. The number of hospital days after surgery was 47.6 ± 39 in the anterior group and 50.8 ± 27.1 in the posterior group. The total number of hospital stays was 58.9 ± 43.4 in the anterior group and 66.8 ± 43.6 in the posterior group (Table 2).

The number of unplanned surgery was less often in the anterior group (n = 4, 5.1%) than in the posterior group (n = 30, 34.1%) (*p* < 0.05). However, neurological deficit and/or pain aggravation were not different between the two groups. Approach-related complications tended to be more often in the anterior group than in the posterior group, although they showed no significant difference: 6.4% in the anterior group and 1.1% in the posterior group (*p* = 0.067). There were no significant differences in radiologic outcomes such as instability, sagittal vertebral axis, or lumbar lordosis. One-year ambulation ability after surgery showed no significant difference between the two groups either (*p* > 0.05) (Table 4).

## 4. Discussion

Surgical treatment of spinal infection has evolved alongside the development of various surgical approaches; however, the optimal approach remains controversial in the literature. In our study, unplanned surgery was less frequent in the anterior group than in the posterior group. Similarly, Chen et al. reported that combined anterior and posterior approaches were associated with a lower incidence of infection recurrence and revision surgery compared with other approaches [8].

On the other hand, one study reported that a posterior-only approach is an effective method for achieving infection control and spinal stability, thereby facilitating early mobilization [15]. In addition, it has been reported that a posterior-only approach does not increase the risk with respect to infection control in single-level lumbosacral infectious spondylitis [8]. Another recent study has reported that the posterior-only approach is suitable, especially for patients with medical comorbidities who have less tolerance to long anesthesia time and substantial blood loss [16]. As mentioned in previous studies, the posterior-only approach might be a better option in the presence of a single segment or medical comorbidity. However, based on our findings, we still recommend an anterior approach with or without posterior instrumentation for most patients, as it demonstrated superior overall outcomes [8,16].

Approach-related complications tended to be more frequent in the anterior group than in the posterior group, although the difference was not statistically significant. In the anterior group, two vascular injuries, one diaphragm injury, and one incisional hernia occurred, whereas one neurogenic deficit was observed in the posterior group. Both vascular injuries involved accidental tearing of the common iliac vein and were successfully repaired by a vascular surgeon without permanent sequelae. The diaphragm injury was repaired by a cardiothoracic surgeon, and the patient recovered with standard postoperative care. The incisional hernia occurred at the anterior superior iliac spine bone graft site and was surgically repaired by the general surgery department. Although complications were numerically more frequent in the anterior group, all cases resolved without long-term functional impairment. In contrast, the posterior-only group had one case of cauda equina syndrome. The dura was friable and severely adherent due to infection and was inadvertently torn during surgical manipulation. Despite intraoperative nylon repair, the patient experienced persistent fecal incontinence and decreased anal sphincter tone, although gradual neurological improvement has been observed.

Yaldiz et al. reviewed 39 patients treated with an anterior approach and reported that this approach appeared to be a safe method with a relatively low rate of complications [17]. Major venous vessel injury occurred in two cases during dissection, and other perioperative complications included left iliac artery occlusion. In contrast, another study suggested that the anterior approach may be associated with increased risk due to prolonged operative time, greater blood loss, and a higher likelihood of complications such as postoperative ileus, as well as retrograde ejaculation [18]. Nevertheless, the authors concluded that the anterior approach remained an acceptable and safe option. Since 2010, the adoption of anterior approaches, including direct lumbar interbody fusion (DLIF) and oblique lumbar interbody fusion (OLIF), has increased substantially at our institution, with the number of anterior procedures rising more than fivefold. Most complications in our cohort occurred during the early 2010s. As surgical experience with anterior approaches increased, the incidence of complications markedly declined.

A small number of infection-related deaths occurred during the study period, predominantly in patients with severe systemic comorbidities and critical medical conditions. However, the clinical circumstances and mechanisms of these events were heterogeneous, including septic shock, end-stage renal disease-related deterioration, and postoperative respiratory failure. Because of the limited number of events and the heterogeneity of these cases, reliable assessment of a causal relationship between mortality and surgical approach was not feasible. Therefore, infection-related mortality was not included in the primary comparative outcome analysis of the present study, and the findings should be interpreted with caution.

In our results, postoperative correction and alignment did not show any significant difference between anterior and posterior groups. Previous studies have reported that pyogenic spondylitis can destroy the vertebral body and lead to spinal deformity (typically kyphosis). Therefore, early diagnosis and timely intervention are critical to prevent progressive deformity [19]. Lee et al. reported that anterior decompression with radical debridement resulted in significant improvement of kyphotic deformity [20]. Another study showed that an anterior approach, with or without posterior instrumentation, effectively prevented spinal deformity in patients with lumbar pyogenic spondylitis in the subacute phase accompanied by advanced vertebral destruction [21]. Conversely, a recent study demonstrated that a posterior-only approach could achieve sagittal alignment correction in single-level lumbosacral spondylitis [22]. In our cohort, the distribution of involved segments and operative levels was comparable between the anterior and posterior groups, with the majority of cases involving single-level disease in the lumbar spine. This similarity in baseline disease characteristics may partly explain the lack of significant differences in postoperative correction and alignment between the two approaches.

Baseline severity of infection is another important factor that may influence both the selection of surgical approach and treatment outcomes in spinal infections. Radiologic indicators such as the extent of infected spinal segments, the presence of epidural abscess, and the degree of vertebral body destruction are commonly considered markers of infection severity. In the present study, we evaluated these baseline infection characteristics, including infected spinal level, number of infected segments, presence of epidural abscess, and anterior vertebral body collapse. Our results demonstrated that these radiologic parameters were comparable between the anterior and posterior groups. These findings suggest that differences in baseline infection severity were unlikely to fully account for the observed differences in clinical outcomes between the two groups.

The subject of one- or two-stage surgery remains controversial. In our study, two-stage surgery (63.4%) was more common than one-stage surgery (36.6%). The authors determined whether one- or two-stage surgery in consultation with a multidisciplinary team, considering patients’ general conditions before surgery and the size of the surgery. One-stage surgery is a safe and efficient way to control infection and stabilize affected segments, allowing for early mobilization of patients [23]. Patients who undergo a one-stage surgery also show fast neurological recovery and return to their preoperative activity with good functional outcomes [24]. On the contrary, Fukuta et al. have reported the success of a series of pyogenic vertebral osteomyelitis treated with a two-stage surgery, suggesting that a two-stage operation with a convalescence period bridging two surgeries might have merits such as shorter operation time, less blood loss, and a higher level of safety for patients with a poorer general health condition as compared to a one-stage operation [25].

For upper thoracic spine levels such as T3–4, an anterior approach may be associated with increased technical difficulty and procedural risk due to the close proximity of vital structures, including the aortic arch, superior vena cava, brachiocephalic artery and vein, esophagus, and trachea. The narrow thoracic inlet and the presence of the lungs further restrict the surgical corridor, increasing the potential for major vascular injury, airway compromise, and pulmonary complications. Accordingly, in selected upper thoracic cases, a posterior approach may be preferentially considered based on anatomical constraints, although anterior-based strategies remain advantageous in most other spinal levels.

This study has some limitations. First, the sample size was relatively small, with only 166 cases included, which may limit the generalizability of the findings. Second, many factors commonly considered in the treatment of spinal infection, such as medical cost, operative time, and transfusion requirements, were not analyzed in this study. Nevertheless, anterior approaches with or without posterior instrumentation are traditionally associated with higher medical costs, longer operative times, and increased transfusion volumes. Third, this was a retrospective study without randomization or comparison with alternative treatment strategies, which may introduce selection bias related to surgical approach selection.

Because no standardized guideline currently exists regarding the optimal surgical approach for pyogenic spondylitis, surgical decision-making in actual clinical practice is often influenced by multiple factors, including anatomical involvement, patient comorbidity, and individual surgical judgment. However, treatment decisions in the present study were made through multidisciplinary discussion, and baseline clinical and radiologic characteristics were generally comparable between the two groups. In this context, comparative clinical studies may provide useful evidence to support surgical decision-making.

Despite these limitations, the present study provides a real-world comparison of treatment outcomes according to surgical approach in routine clinical practice. The potential benefits of anterior approaches with or without posterior instrumentation should be further validated in large-scale, multicenter prospective studies.

## 5. Conclusions

An anterior approach with or without posterior instrumentation may represent a more favorable surgical strategy than a posterior-only approach, as it was associated with lower rates of treatment failure, particularly with respect to revision surgery. Although infection-related mortality was higher in the anterior approach group, these events appeared to be primarily attributable to patients’ preoperative systemic conditions rather than the surgical approach itself. Furthermore, approach-related complications in the anterior group resolved without permanent sequelae.

Taken together, these findings suggest that an anterior approach with or without posterior instrumentation should be carefully considered as a primary surgical option in the management of pyogenic spondylitis.

## Figures and Tables

**Table 1:** Demographic data of subjects in the anterior group and the posterior group.

Variable	Anterior (n = 78)	Posterior (n = 88)	*p*-Value
Age, mean (SD)	66.86 ± 12.2	67.16 ± 12.16	0.875
Sex			0.528
Male	48 (61.54%)	49 (55.68%)	
Female	30 (38.46%)	39 (44.32%)	
Smoking	20 (25.64%)	23 (26.14%)	>0.99
BMI			0.455
BMI < 25	47 (60.26%)	59 (67.05%)	
BMI ≥ 25	31 (39.74%)	29 (32.95%)	
Comorbidity			
Hypertension	43 (55.13%)	48 (54.55%)	>0.99
Diabetes mellitus	32 (41.03%)	26 (29.55%)	0.166
Liver cirrhosis	3 (3.85%)	1 (1.14%)	0.343
Hemodialysis	11 (14.1%)	5 (5.68%)	0.116
Malignancy	6 (7.69%)	13 (14.77%)	0.236
ASA			0.674
1	11 (14.1%)	9 (10.23%)	
2	46 (58.97%)	59 (67.05%)	
3	19 (24.36%)	19 (21.59%)	
4	2 (2.56%)	1 (1.14%)	

**Table 2:** Clinical characteristics of subjects in the anterior group and the posterior group.

Variable	Anterior (n = 78)	Posterior (n = 88)	*p*-Value
Infection type			0.225
Primary infection	43 (55.13%)	43 (48.86%)	
Secondary infection without metal instrument	9 (11.54%)	19 (21.59%)	
Secondary infection with metal instrument	26 (33.33%)	26 (29.55%)	
Operation type			
One-stage	26 (36.61%)		
Two-stage	45 (63.38%)		
Microbes			
MSSA	3 (3.85%)	7 (7.95%)	0.338
MRSA	7 (8.97%)	8 (9.09%)	>0.99
*S. epidermidis*	12 (15.38%)	11 (12.5%)	0.755
Other staphylococci	6 (7.69%)	4 (4.55%)	0.518
*Enterococcus* species	6 (7.69%)	3 (3.41%)	0.308
*Streptococcus* species	3 (3.85%)	6 (6.82%)	0.503
*E. coli*	2 (2.56%)	5 (5.68%)	0.449
*Enterobacter* species	0 (0%)	1 (1.14%)	>0.99
*K. pneumonia*	2 (2.56%)	0 (0%)	0.219
Others	7 (8.97%)	6 (6.82%)	0.821
No growth	30 (38.46%)	38 (43.18%)	0.646
Hospital days after surgery (day)	47.58 ± 39	50.75 ± 27.07	0.110
Total hospital stay (day)	58.85 ± 43.4	66.83 ± 43.6	0.077

**Table 3:** Baseline radiologic characteristics of spinal infection.

Variable	Anterior (n = 78)	Posterior (n = 88)	*p*-Value
Infected spinal level			0.9354
T spine level (≥T10)	3 (3.85%)	5 (5.68%)	
T-L junction level (T10-L2)	10 (12.82%)	10 (11.36%)	
L spine level (L2-5)	51 (65.38%)	56 (63.64%)	
L-S (L5-S1)	14 (17.95%)	17 (19.32%)	
Number of infected segments			0.5448
1	52 (66.67%)	64 (72.73%)	
2	16 (20.51%)	17 (19.32%)	
3	8 (10.26%)	4 (4.55%)	
4	2 (2.56%)	3 (3.41%)	
5	0 (0%)	0 (0%)	
Epidural abscess			0.3085
Absent	39 (50%)	36 (40.91%)	
Present	39 (50%)	52 (59.09%)	
Anterior vertebral body height loss			0.89
<50%	68 (87.18%)	75 (85.23%)	
≥50%	10 (12.82%)	13 (14.77%)	

**Table 4:** Clinical outcomes of subjects in the anterior group and the posterior group.

Variable	Anterior	Posterior	OR (95%CI)	*p*-Value
	(n = 78)	(n = 88)		
Unplanned surgery	4 (5.13%)	30 (34.1%)	8.9739 (2.95–27.29)	<0.001
Neurological deficit/Pain aggravation	11 (14.1%)	8 (9.0%)	0.61 (0.22–1.70)	0.342
Approach related complication	4 (6.4%)	1 (1.1%)	0.0763 (0.005–1.200)	0.067
Radiologic outcome				
Postop 1Y instability (translation)	9 (16.1%)	11 (23.9%)	0.39 (0.10–1.51)	0.175
Postop 1Y instability (angulation)	1 (1.8%)	1 (2.2%)	1.20 (0.07–19.72)	0.898
Postop 1Y SVA	23 (29.4%)	6 (54.6%)	0.48 (0.08–2.93)	0.433
Postop lumbar lordosis	12 (17.1%)	19 (26.8%)	1.90 (0.75–4.83)	0.174
Ambulation ability				
0	42 (53.9%)	46 (52.3%)	Reference	
1	10 (12.8%)	6 (6.8%)	0.51 (0.16–1.57)	0.239
2	3 (3.9%)	11 (12.5%)	3.13 (0.80–12.26)	0.102
3	1 (1.3%)	5 (5.7%)	40.27 (0.37–10.66)	0.182
4	7 (9.0%)	9 (10.2%)	1.11 (0.38–3.31)	0.840
5	5 (6.4%)	3 (3.4%)	0.51 (0.11–2.34)	0.386
6	10 (12.8%)	8 (9.1%)	0.68 (0.24–1.95)	0.474

SVA = Sagittal vertical axis.

## Data Availability

The data presented in this study are available from the corresponding author on reasonable request. The data are not publicly available due to privacy and ethical restrictions.
